# Effect of extraction time on content, composition and sensory perception of proanthocyanidins in wine‐like medium and during industrial fermentation of Cabernet Sauvignon

**DOI:** 10.1002/jsfa.10189

**Published:** 2020-02-04

**Authors:** Klemen Lisjak, Zorica Lelova, Uroš Žigon, Špela Velikonja Bolta, Pierre‐Louis Teissedre, Andreja Vanzo

**Affiliations:** ^1^ Department of Fruit Growing, Viticulture and Oenology and Central Laboratory Agricultural Institute of Slovenia Ljubljana Slovenia; ^2^ Tikveš winery Skopje Macedonia; ^3^ Frutarom Etol d.o.o Škofja Vas Slovenia; ^4^ Université de Bordeaux, ISVV, EA 4577, Oenologie Villenave d'Ornon France

**Keywords:** proanthocyanidins, extraction, grape seeds, grape skins, industrial fermentation

## Abstract

**BACKGROUND:**

The research objectives focused on the extraction of grape tannins during extended maceration. Skins and seeds were extracted separately in a wine‐like medium. In parallel, the same grapes were fermented in industrial tanks. The content and structural characteristics of extractable proanthocyanidins (PAs) were determined spectrophotometrically and using UHPLC‐DAD‐MS/MS, respectively. Skin, seed extracts and fermented wines were characterized in chemical and sensorial terms after different extraction durations.

**RESULTS:**

The extraction of high molecular‐weight PAs (HMWPs) from seeds increased for up to 20 days, whereas low molecular‐weight PAs (LMWPs) reached a plateau earlier. The extraction of HMWPs and LMWPs from skins reached a maximum at the first sampling. Sensory evaluation confirmed greater astringency and bitterness of seed extracts with increasing time. Neither seed nor skin extracts differed statistically in terms of the mean degree of polymerization (mDP) and percentage of galloylation (%G) on different extraction days (except for seeds at the first sampling). During industrial maceration, HMWPs and LMWPs increased up to 12.7% alcohol (9 days of maceration); thereafter, the increase was not significant, whereas the mDP, %G and percentage of prodelphinidins did not significantly change after 11.4% alcohol. There were positive correlations with the wine astringency and PA content.

**CONCLUSION:**

Looking at both simulated and industrial maceration, it can be concluded that, with a longer maceration time, the increase in HMWP content was more evident than PA structural changes. The increasing content of tannins from seeds played an important role in the greater astringency and bitterness of Cabernet Sauvignon macerated at length. © 2019 The Authors. *Journal of The Science of Food and Agriculture* published by John Wiley & Sons Ltd on behalf of Society of Chemical Industry.

## INTRODUCTION

Grape proanthocyanidins (PAs), also known as condensed tannins, impair some important wine sensorial properties, such as color, bitterness, astringency, turbidity, and ageability. They are present in the skins, seeds, and stems of grapes.^1^, ^2^ Grape‐seed PAs are composed of the flavan‐3‐ol monomers catechin, epicatechin, and epicatechin gallate.^3^, ^4^, ^5^ Grape‐skin PAs are composed of catechin, epicatechin, epigallocatechin, and gallocatechin^4^, ^5^, ^6^ and a low proportion of galloylated units.^5^ Skin PAs differ from those in seeds due to the presence of prodelphinidins (polymeric tannins composed of epigallocatechin and gallocatechin), a higher mean degree of polymerization (mDP), and a smaller proportion of galloylated subunits.^5^, ^7^ Epigallocatechin units and epicatechin gallate units can therefore be used as markers of PAs from skins and seeds respectively.^8^

The organoleptic differences between grape seed and skin PAs largely depend on their structural characteristics. Low molecular‐weight PAs (LMWPs) are both bitter and astringent,^9^ whereas high molecular‐weight PAs (HMWPs) are astringent.^10^, ^11^ Chira *et al*.^12^ extracted PAs from lyophilized and ground seeds and skins of Merlot and Cabernet Sauvignon with strong organic solvents. After fractionation, they diluted the monomeric / oligomeric and polymeric fractions of PAs in a wine‐like medium at the same concentration. The seed and skin monomeric / oligomeric fraction was sensorially characterized as slightly astringent and the polymeric seed fraction as tannic, whereas the polymeric skin fraction was characterized as rather mellow. The astringency of red wines intensifies with a higher mDP and a higher percentage of galloylation (%G),^13^, ^14^ and a higher PA content.^15^ The lower proportion of galloylated subunits and the presence of prodelphinidins may be the reason why skin PAs are traditionally regarded by oenologists as pleasanter and softer than seed PAs.^14^

The grape variety affects both the amount and structure of PAs in seeds and skins.^4^, ^16^ Furthermore, vintage and terroir were shown to be important factors in terms of the structural characteristics of PAs in skins and seeds,^17^ whereas the harvesting date and maceration management are important decisions for oenologists, producing red wines with distinctive styles. During maceration it is difficult to distinguish whether PAs have been extracted from the skins or seeds. However, the origin and structural characteristics of extractable PAs are important for organizing proper cap management and optimizing extraction. Extractable PAs from separated skin and seed tissues in wine‐like medium have been investigated in different grape varieties in several studies.^4^, ^18^, ^19^, ^20^, ^21^, ^22^ These studies aimed to provide variety‐specific information about the content and / or structural characteristics of flavan‐3‐ols extractable from skins and seeds. It was found that skin PAs are more readily extractable in wines or wine‐like mediums, whereas extraction from seeds requires longer maceration.^19^, ^22^, ^23^, ^24^ Mattivi *et al*.^4^ reported that both seed and skin extracts in wine‐like medium were mainly rich in monomers and small oligomers (mDP of less than 8) and that the majority of extractable free flavanols in grapes were located in the seeds. Selective extraction of seeds and skins in a wine‐like medium may also have some limitations. It was recently reported that the content and composition of tannins in seed and skin extracts were modified due to their adsorption on mesocarp material.^18^ Another option might be the use of an analytical method for measuring PA extraction from seeds and skin directly in wines.^25^ In this study it was found that seed and skin PA extension subunits were different, their composition did not vary with extraction time, and the proportion of skin PAs declined during fermentation.^25^ Direct methods also showed some limitations, such as PA composition differences between varieties, and the different potential of skin and seed PAs to become oxidized.

The aim of this study was to investigate the influence of maceration time on the content and structural characteristics of seed and skin PAs extractable in wine. The seeds and skins of mature Cabernet Sauvignon grapes were extracted separately in a wine‐like medium, which was used to simulate fermentative maceration. In parallel, grapes from the same vineyard were fermented in large stainless‐steel fermenters. Skin and seed extracts and fermented wines were analyzed on different days to cover the period of maceration. The LMWP and HMWP content in skin, seed extracts, and macerated wines was analyzed spectrophotometrically. The structural characteristics of extractable PAs, such as mDP, percentage of galoyllation (%G), and percentage of prodelphinidin (%P) were determined with UHPLC‐DAD‐MS/MS. Seed and skin wine‐like extracts and fermented wines were also characterized by a trained panel of professional wine tasters to determine their sensorial properties (in terms of color intensity, astringency and bitterness).

## MATERIAL AND METHODS

### Chemicals

Pure HPLC grade analytical standards (+)‐catechin, (−)‐epicatechin, (−)‐gallocatechin, (−)‐epigallocatechin, and (−)‐epicatechin gallate were obtained from Sigma (Steinheim, Germany).

### Grape sampling

Grapes from cv. Cabernet Sauvignon (*Vitis vinifera* L.) were sampled in 2016 in the ‘Demir Kapija’ vineyard in Macedonia (*X* = 41.4115; *Y* = 22.2298; Huglin Index was 2839). The vineyard was planted in 1990 on silty loam to loam soil with a low water‐holding capacity. It is situated at 140 m a.s.l., with the rows oriented north–south. Due to low total rainfall from April to September (293 mm), irrigation took place during the vegetation phase, according to standard protocols used in the winery. The grapes were manually harvested at full technological maturity with the following physicochemical results: Brix 24.5°, pH value of 3.9, total acidity of 5.3 g L^−1^ as tartaric acid equivalents and malic acid content of 1.8 g L^−1^. Approximately 20 kg of grape bunches from various parts of the vineyard were representatively sampled on the day of harvest and brought to the laboratory for extraction studies in model wine solution. The grapes were cooled overnight at 4 °C.

### Extraction of polyphenols from skins and seeds in a wine‐like medium

To investigate the composition of the flavanol fraction, which is extractable from grapes and passes into the wine, selective extraction of phenols from the skin and seeds of grape berries simulating the red wine maceration process was used, as described.^26^ Berries were separated from the stems with scissors and randomly divided into 18 units, each weighing 200 g (3, 5, 7, 10, 15, and 20 days' extraction in triplicate). To avoid changes in ratio between solids and liquids during extraction, each unit was treated separately. Skins and seeds were separated and extracted at 30 °C in a 200 mL solution consisting of ethanol:water (12 : 88 v/v), 100 mg L^−1^ SO_2_, 5 g L^−1^ tartaric acid and with a pH value adjusted to 3.2 (with NaOH). To minimize PA oxidation, the solutions were flushed with nitrogen and extraction was carried out in the dark. The extracts were manually shaken once a day. After 3, 5, 7, 10, 15, or 20 days, the seeds and skins were removed from the hydroalcoholic solution. The skin extract was centrifuged for 10 min at 3500×*g* and the extraction volume was adjusted. Both seed and skin extracts were transferred into dark glass bottles, flushed with nitrogen, and stored at 4 °C until spectrophotometric, UHPLC‐DAD‐MS/MS, and sensorial analysis was carried out. Chemical and sensorial analysis was performed within 3 months.

### Large‐scale vinification

Cabernet Sauvignon berries were mechanically separated with a destemmer (Selectiv' Process Winery, Pellenc, Pertius, France) into three 7 ton concrete fermenters allowing temperature control. The whole berries were macerated and fermented. All the fermenters were inoculated with yeasts (20 g dry wt hL^−1^ Zymaflore F15, Laffort, Bordeaux, France) and nutrients (20 g wt hL^−1^ Fermaid O, Lallemand, Montreal, Canada) and the pomace was punched down twice a day during active fermentation and once a day during prefermentation. *Delastage* (‘rack‐and‐return’ of fermenting juice) was performed three times during maceration. Cold prefermentation at 10–15 °C lasted 2 days and thereafter the temperature was 25 °C. Alcoholic fermentation started on the third day. After 15 days of maceration, free run wine was decanted and the pressings were separated. Wine samples were collected from the middle of the fermenter 3, 5, 7, 9, 11, 13, and 15 days following punch down, centrifuged at 4 °C for 10 min at 3000×*g* and frozen at −20 °C until analysis, which was carried out 6 months later.

### Spectrophotometric analysis

Analysis was performed with a Varian Cary 100 spectrophotometer (Agilent Technologies Inc., Palo Alto, CA, USA) as described.^27^ Polar compounds such as sugars, organic acids, amino acids, and free SO_2_, which could interfere with assays, were removed with a clean‐up of grape extracts and wine using Sep‐Pak C‐18 columns (0.5 g, Waters).

Total polyphenols (TPs) were assessed by a reduction of Folin–Ciocalteu reagent to blue pigments caused by phenols in alkaline solution. The concentrations of total polyphenols were determined by means of a calibration curve as (+)‐catechin in mg kg^−1^ of grape fresh weight (FW) or mg L^−1^ of wine.

High molecular‐weight proanthocyanidins were evaluated by transformation into cyanidin and expressed in mg kg^−1^ of cyanidin chloride grape FW or mg L^−1^ of wine. The method is a highly specific assay, which provides a good evaluation of the total amount of PAs, and it is mainly linked to variations in the HMWP corresponding to at least 5 units.^28^

Low molecular‐weight proanthocyanidins were determined by their reaction with vanilin. The vanillin index methods provide an estimation of free C6 and C8 for catechins and for proanthocyanidins. This index decreases with an increase in polymerization, because C6 and C8 are mainly involved in polymerization bonds. The method therefore provides a good estimation of monomers and a low degree of polymerized flavanols corresponding to two to four units. Low molecular‐weight proanthocyanidins were evaluated as (+)‐catechin in mg kg^−1^ of grape FW or mg L^−1^ of wine.

Total anthocyanins (TAs) were determined on the basis of maximal absorbance in the visible range (536–542 nm). They were quantified in mg kg^−1^of grape FW by assuming an average absorbance of the mixture of anthocyanins extracted from Cabernet Sauvignon grapes (average MW = 500 Da, *ϵ* = 18 800 M^−1^ cm^−1^ in 70:30:1 ethanol:water:HCl solution).

Color intensity and hue of wines: color intensity is determined and defined as the sum of the absorbance at 420, 520, and 620 nm.^29^ The hue of the wine is defined as the ratio of A420/A520.^29^ This was measured directly at 420, 520, and 620 nm on a 1 mm optical path, multiplying the results by 10.

### Fractionation of proanthocyanidins in seed and skin wine‐like extracts and wines using phloroglucinol

The structural characteristics of PAs, comprising mDP, %G and %P were determined after acid‐catalyzed degradation with phloroglucinol as nucleophilic reagent.^30^, ^31^A slightly modified method was used.^32^ Briefly, 2.5 mL of sample was evaporated under reduced pressure at 40 °C on a Buchi rotavapor to approximately 80% of the initial volume to remove the ethanol fraction. The evaporated sample was quantitatively transferred into a volumetric flask and diluted to 10 mL with MilliQ ultra‐pure water (EMD Millipore, Billerica, MA, USA). The diluted sample was loaded onto a LC18 cartridge (Supelco, St Quentin Fallavier, France) previously conditioned with 50 mL of methanol and 50 mL of Milli Q. The column was washed with 50 mL of MilliQ, dried with nitrogen to remove remaining traces of water and the sample was eluted with 50 mL of methanol. Methanol was evaporated under reduced pressure at 40 °C until dryness, and the sample was redissolved in 1 mL of methanol. A 200 μL aliquot of methanol fraction was mixed with 200 μL of phloroglucinol reagent (0.05 mol L^−1^ of HCl in methanol, 50 g L^−1^ of phloroglucinol (Sigma Aldrich, Steinheim, Germany) and 10 g L^−1^ of ascorbic acid (Merck, Darmstadt, Germany). The mixture was held at 50 °C for 20 min for fractionation. After 20 min, 1 mL of 40 mmol L^−1^ aqueous sodium acetate was added to stop the reaction. Samples were filtered through a 0.22 μm PVDF filter from Millipore (Billerica, MA, USA) and analyzed using UHPLC‐DAD‐MS/MS.

### UHPLC‐DAD‐MS/MS and UHPLC‐DAD‐MS/TOF analysis of flavanols

The reaction products were analyzed with a 1290 infinity UHPLC system coupled to an ultrasensitive DAD detector (G4212A) and a 6460 triple quadrupole mass spectrometer (Agilent Technologies, Santa Clara, CA, USA). For further compound confirmation, some of the samples were injected into UHPLC‐DAD‐TOF/MS (Agilent Technologies) under the same analytical conditions. Samples were kept at 4 °C during analysis and the injection volume was 10 μL. Separation was performed on a 100 mm × 4.6 mm, 3.5 μm column (XTerra RP18, Waters). Flow was set to 0.7 mL min^−1^, mobile phase A was 1% (v/v) aqueous acetic acid and B methanol. Separation was carried out at 40 °C using a linear gradient starting at 5% B for 25 min, from 5% to 20% B in 20 min, from 20% to 32% B in 15 min and from 32% to 100% B in 2 min; 100% B for 5 min, to 5% B in 1 min and 5% B for 4 min. The UV‐visible spectra were recorded from 210 to 400 nm with detection at 280 nm. Total ion chromatograms (TICs) were monitored in negative ion mode using electrospray ionization mass spectrometry (ESI, Jet‐Stream). Nitrogen was used as the carrier gas and the source parameters were: gas temperature 250 °C, gas flow 6 L min^−1^, nebulizer 241 KPa, sheath gas heater 375 °C, sheath gas flow 10 L min^−1^, capillary voltage 3500 V. Identification of flavan‐3‐ols and their phloroglucinol adducts was performed using the molecular ion (M‐H)^−^, which was m/z 289 for catechin and epicatechin; m/z 305 for gallocatechin and epigallocatechin; m/z 441 for epicatechin gallate; m/z 413 for catechin‐ and epicatechin‐phloroglucinol, m/z 429 for epigallocatechin‐phloroglucinol and m/z 565 for epicatechin gallate‐phloroglucinol. Accurate masses were determined with UHPLC‐DAD‐MS/TOF, using (M‐H)^−^, which was m/z 289.0733 for catechin; m/z 289.0727 for epicatechin; m/z 305.0704 for gallocatechin; m/z 441.0825 for epicatechin gallate, m/z 413.0889 for catechin‐phloroglucinol; m/z 413.0909 for epicatechin‐phloroglucinol, m/z 429.0897 for epigallocatechin‐phloroglucinol and 565.0986 for epicatechin gallate‐phloroglucinol. Flavan‐3‐ol monomers were also injected as external standards.

The mDP, %P and %G were estimated using the response factors of PA cleavage products at 280 nm and calculated as described.^32^ The mDP value represented the molar ratio between the sum of all flavan‐3‐ol units produced by phloroglucinolysis and the sum of terminal units.

### Sensory characterization

Sensory characterization of the astringency and bitterness of wine‐like seed and skin extracts and wines was performed with a panel of seven professional wine tasters and oenologists at the winery. They were trained with standard solutions to familiarize them with the terms of astringency and bitterness, as well as the intensity scale. The standard solution for evaluation of bitterness was quinine sulfate (0.05, 0.1, and 0.2 g L^−1^; Merck, Darmstadt, Germany) in 12% ethanol in water. The standard solution for evaluation of astringency was aluminium sulfate (0.5, 1.0, and 2.0 g L^−1^; Alkaloid, Skopje, Macedonia) in 12% ethanol in water. Bitterness and astringency intensities were classified on a 0–7 point scale.^23^ The skin and seed extracts and fermented wines from different days of extraction were characterized with a ranking test, from the least to the most astringent, the least to the most bitter, and the least to most intense color. Samples were served to tasters in a random order and assigned a three‐digit number for identification. To avoid the problem of a carryover effect, at least 30 s time between tasting each sample was considered. They were tasted at room temperature (20 ± 2 °C) in OIV tasting glasses and testing was performed at room temperature in daylight.

### Statistics

Statistical analysis was conducted using the R statistical program and Statistica software. When there was only one fixed factor, a one‐way ANOVA was used. Before analysis, the homogeneity of variance at different fixed factor levels (samples) was checked with the Breusch–Pagan test (R). In the two‐way scenario, where there was also the assessor random factor, besides the fixed sample factor, a linear mixed model was used (nlme package, R). If statistically significant fixed effects (differences in sample means) were confirmed in the selected model (ANOVA, mixed model), Tukey's multiple comparison test followed with the glht (generalized linear hypothesis testing) function in R. This compares all sample pairs (sample means) while controlling the family‐wise error rate (FWER) at a 5% significance level. The data obtained with the ranking test were analyzed with the Friedman sum‐of‐rank test with an expected Friedman statistic value equal to the degrees of freedom (n‐1). If the Friedman test was significant (*P* < 0.05), the LSD (least significant difference) value was calculated to assess all pair differences in sum ranks (ISO 8587;2006) at a 5% significance level.

## RESULTS AND DISCUSSION

### UHPLC‐DAD‐MS/MS method validation

The UHPLC‐DAD‐MS/MS chromatograms of flavan‐3‐ol monomers and their respective phloroglucinol adducts in Cabernet Sauvignon grape seed and skin extracts are presented in the supporting information (Fig. S1). The method was validated for repeatability, reproducibility, and uncertainty (supporting information, Table S1). Repeatability and reproducibility were determined over a 10 day period; two samples of Merlot wine (2013) were prepared each day. Standard deviation of repeatability and standard deviation of reproducibility were calculated. Dispersion of results was checked with Cochran's test and outliers with Grubbs' test (ISO 5725‐2:1994). Uncertainty of repeatability and uncertainty of reproducibility were calculated by multiplying the standard deviation of repeatability and the standard deviation of reproducibility by Student's *t* factor with 9° of freedom and a 95% confidence level (*t*_95; 9_ = 2262). *U*_*r*_ = *t*_95; 9_ × *s*_*r*_; *U*_*R*_ = *t*_95; 9_ × *s*_*R*_.

### Phenolic content in skin and seed wine‐like extracts

**Figure 1:**
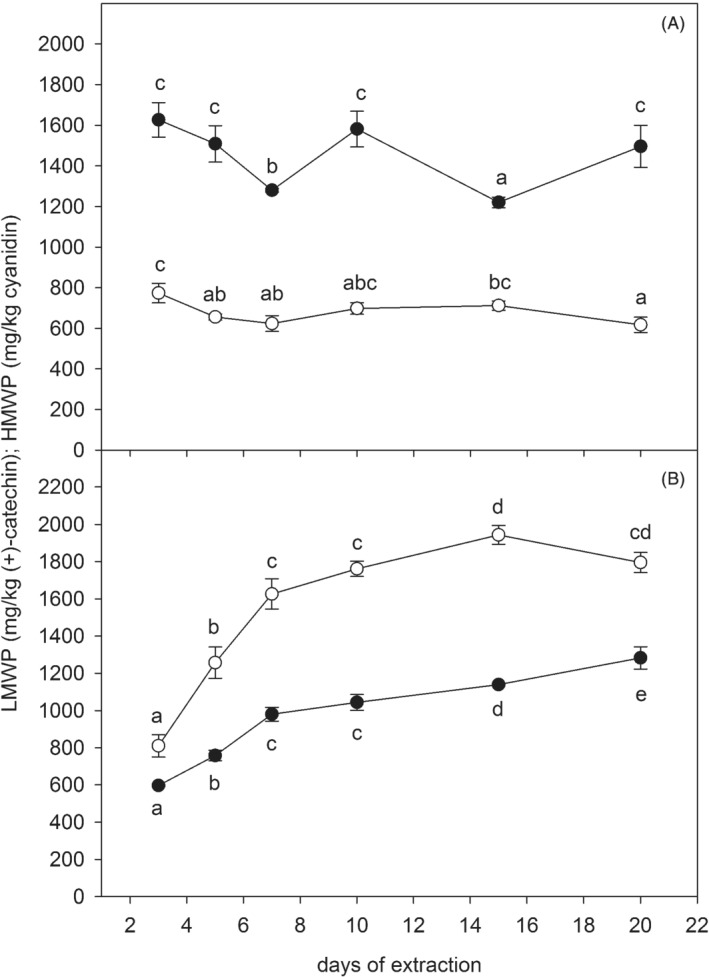
Content of extractable low and high molecular weight proanthocyanidins (expressed in mg kg^−1^ of grape FW) after different extraction durations for A (skins) and B (seeds) in a wine‐like medium (LMWPs: hollow symbols 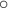, HMWPs: solid symbols 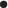). Error bars represent the standard deviation of three replicates for each observation (n = 3). Different letters indicate statistically significant differences between extraction days (Tukey's HSD, *P* < 0.05).

The content of LMWPs, HMWPs, total anthocyanins and total polyphenols was determined spectrophotometrically in skin and seed wine‐like extracts in triplicate after different extraction times. The HMWP and LMWP analytical data allowed us to show the differences in the reactivity of grape PAS for both assays, which suggested different degrees of polymerization. However, for better quantification of these differences, advanced analytical techniques such as liquid chromatography connected to ultraviolet‐visible and mass spectrometry should be used. In general, skins contained a higher proportion of HMWPs (Fig. 1(a)), whereas seeds contained a higher proportion of LMWPs (Fig. 1(b)). This is in agreement with findings of higher mDP in skins than in the seeds of Cabernet Sauvignon grapes. The results showed that both LMWPs and HMWPs were rapidly extracted from skins in a wine‐like medium. At the first sampling, i.e. after 3 days of extraction, the peak level was already reached (Fig. 1(a)). Others have also reported maximum release of flavanols from skins after 24 h of maceration in 12.5% ethanol, whereas maximum flavanol extraction from seeds was observed after 2–3 weeks of maceration.^19^ As regards seed extracts, the HMWP content significantly increased throughout the extraction period, up to 20 days, whereas the LMWP content reached a peak after 15 days (Fig. 1(b)). The method for determining LMWPs comprises free flavan‐3‐ols and PA oligomers, which, according to our results, were extracted faster than polymers (Fig. 1(b)). This is consistent with the results of Hernández‐Jiménez *et al*.,^33^ who found that the percentage of monomeric compounds from seeds decreased with maceration time (they are readily extracted at the beginning of maceration) and the percentage of polymeric PAs increased. In our study, the HMWP extraction rate from seeds was faster up to 7 days than from 7 to 20 days. After 7 days, a linear increase was found (Fig. 1(b)). This is consistent with a study in which it was shown that the presence of 10% or 15% alcohol increased the initial extraction rate of PAs from seeds, and after 6 days the extraction rate was linear and nearly identical, even in the absence of alcohol.^33^

The temperature of extraction may be another factor affecting the extraction rate, as shown by Lerno *et al*.^34^ In our study the temperature of extraction was 30 °C throughout extraction, and in addition to ethanol, this could accelerate the initial rate of both LMWP and HMWP extraction from skins and seeds.

The average distribution (n = 3 for each day) of extractable LMWPs and HMWPs in seeds and skins after different extraction times in a wine‐like medium is shown in the supporting information, in Figs S3 and S4 respectively. It was found that 51% of total LMWPs were extracted from seeds and 49% from skins after 3 days of extraction, compared to 74% and 25% after 20 days respectively. The higher proportion of LMWPs in seed extracts might be the consequence of higher amounts of monomers in the seeds.^4^ As regards HMWPs, it was found that 27% of the total was extracted from seeds and 73% from skins after 3 days, compared to 46% and 54% respectively after 20 days. The skin PA extraction rate exceeded that of seeds, as also demonstrated by others.^19^, ^22^, ^24^

**Figure 2:**
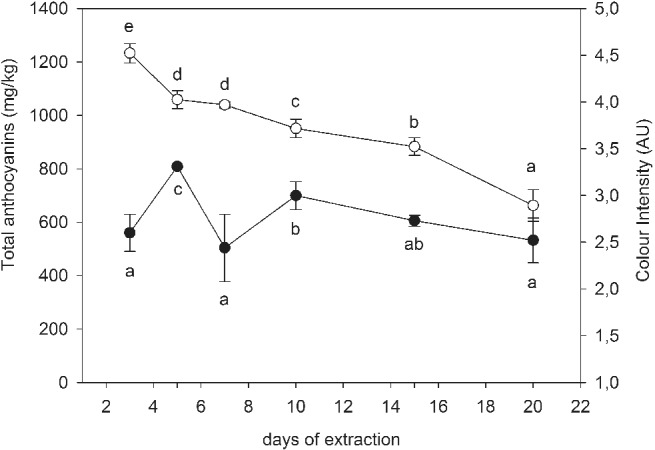
Average content of total extractable anthocyanins (in mg kg^−1^of grape FW: hollow symbols 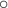) and average color intensity (solid symbols 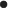) after different extraction durations for skins and seeds in a wine‐like medium. Error bars represent the standard deviation of the measurements for each observation. Different letters indicate statistically significant differences between extraction days (Tukey's HSD. *P* < 0.05).

The total content of extractable anthocyanins in skin extracts reached a maximum after 3 days of extraction and gradually decreased over time (Fig. 2). The lowest concentration was detected after 20 days of extraction and significantly differed from other extraction days. Lerno *et al*.^34^ showed a decrease in malvidin‐3‐*O*‐glucoside in extended maceration at higher (35 °C) temperature. However, color intensity was stable throughout extraction and there were no significant differences between 3, 15, and 20 days of extraction. Stabilization of color intensity could be due to the formation of stable polymeric pigments deriving from the incorporation of anthocyanins in tannins.^35^

The average content of extractable total polyphenols (mg kg^−1^ of grape fresh weight) after different days of extraction from skins and seeds in wine‐like medium is shown (supporting information, Fig. S2 **)**. Initially, i.e. after 3 and 5 days of extraction, the total polyphenol content in skin extracts was higher than in seeds. However, from there on, total polyphenol content was higher in seed extracts. After longer maceration, the proportion of seed polyphenols was higher than in skins. This is in agreement with the results obtained from spectrophotometric analysis of LMWPs and HMWPs.

### Structural characteristics of seed and skin proanthocyanidins in wine‐ like extracts

The structural characteristics of PAs extractable on different days from skins and seeds in wine‐like medium, i.e. mDP, %G and %P, are presented in Table 1. Skin‐based PAs contained the flavan‐3‐ol subunits catechin, epicatechin, gallocatechin, epigallocatechin, and epicatechin‐gallate whereas seed‐based PAs contained catechin, epicatechin, and epicatechin‐gallate (Fig. S1, supporting information).

**Table 1:** Mean degree of polymerization (mDP), percentage of galloylation (%G) and percentage of prodelphinidins (%P) in skin and seed wine‐like extracts. Each day represents the average of three repetitions

	Skin extract			Seed extract	
Days of extraction	mDP	%P	%G	mDP	%G
3	13.0 a	35.2 a	8.6 a	3.6 a	42.0 a
5	14.4 a	36.7 a	7.5 a	5.0 bc	48.8 c
7	15.7 a	36.4 a	7.1 a	4.7 b	46.2 b
10	16.6 a	39.7 a	5.8 a	5.2 c	49.6 c
15	13.5 a	36.7 a	6.1 a	5.1 c	47.6 bc
20	19.9 a	33.5 a	8.2 a	5.2 c	47.7 bc

Different letters within each column indicate significant differences between extraction days (Tukey's HSD, *P* < 0.05).

After 20 days of extraction in wine‐like medium, skin PAs had mDP four times higher and a proportion of galloylated subunits six times lower than seed PAs, while %P was 33.5% (Table 1). Higher mDP was also observed in Cabernet Sauvignon skin PAs than seed PAs by Rinaldi *et al*.,^21^ after 5 days of extraction in wine‐like medium and by Chira *et al*.^17^ after extraction by means of strong solvents. On the other hand, Mattivi *et al*.^4^ analyzed Cabernet Sauvignon (n = 4) skin and seed PAs with thioacidolysis after 5 days of extraction in a wine‐like medium and found an average mDP of 3.1 and 3.2 for skins and seeds respectively.

The percentage of prodelphinidins (%P) in skins ranged from 33.5–39.7 after a different number of days' extraction in wine‐like medium. A high proportion of prodelphinidins in the skins of Cabernet Sauvignon has also been observed by others after extraction in strong organic solvents.^6^, ^23^ In contrast, others have found 16.2%P in Cabernet Sauvignon skins, with mDP and %G of 19.3 and 7.4 respectively, again following extraction in organic solvents.^17^

In our observations there were no significant differences in structural characteristics (mDP, %G and %P) of skin extracts throughout the extraction period. This is in accordance with Peyrot des Gachons and Kennedy,^25^ who showed that the composition of seed and skin PA extension subunits did not vary with extraction time. As regards seed extracts, a significant increase in mDP and %G from 3 to 5 days of extraction was noticed. However, there were no significant structural differences between 5, 7, 10, 15 and 20 days. Similar results were also found by Hernández‐Jiménez *et al*.^33^ who observed no changes in mDP and in the proportion of galloylated PAs after extraction of seeds for between 2 and 10 days in different ethanol : water solutions.

### Sensory characterization of skin and seed wine‐like extracts

To compare the sensory characteristics of skin and seed extracts, a rank test was performed. The results are presented in Table 2. Skin extracts showed no significant differences in sensory attributes related to color intensity, astringency, and bitterness on different days of extraction. As regards color intensity, the sample after 5 days of extraction in the wine‐like medium was ranked highest, but was not significantly different from 3, 15, and 20 days of extraction. The highest color intensity after 5 days was also determined spectrophotometrically (Fig. 2).

**Table 2:** Ranking totals (n = 7 tasters, intensity scale 0–7 points) for sensory attributes regarding color, astringency and bitterness of skin and seed extracts on different extraction days

	Skin extract			Seed extract	
Days of extraction	Color intensity	Astringency	Bitterness	Astringency	Bitterness
3	21 ab	19 ab	19 ab	6 a	11 a
5	26 b	20 ab	12 a	14 ab	10 a
7	14 a	18 ab	24 b	20 bc	19 ab
10	14 a	11 a	16 ab	18 bc	20 ab
15	19 ab	20 ab	19 ab	25 c	24 b
20	17 ab	23 b	21 ab	28 c	27 b

Different letters indicate statistically significant differences between extraction days (LSD 5%).

On the other hand, seed extracts showed there was a trend for astringency and bitterness to increase with extraction time. After 15 and 20 days, seed astringency and bitterness were significantly higher than after 3 and 5 days of extraction.

**Figure 3:**
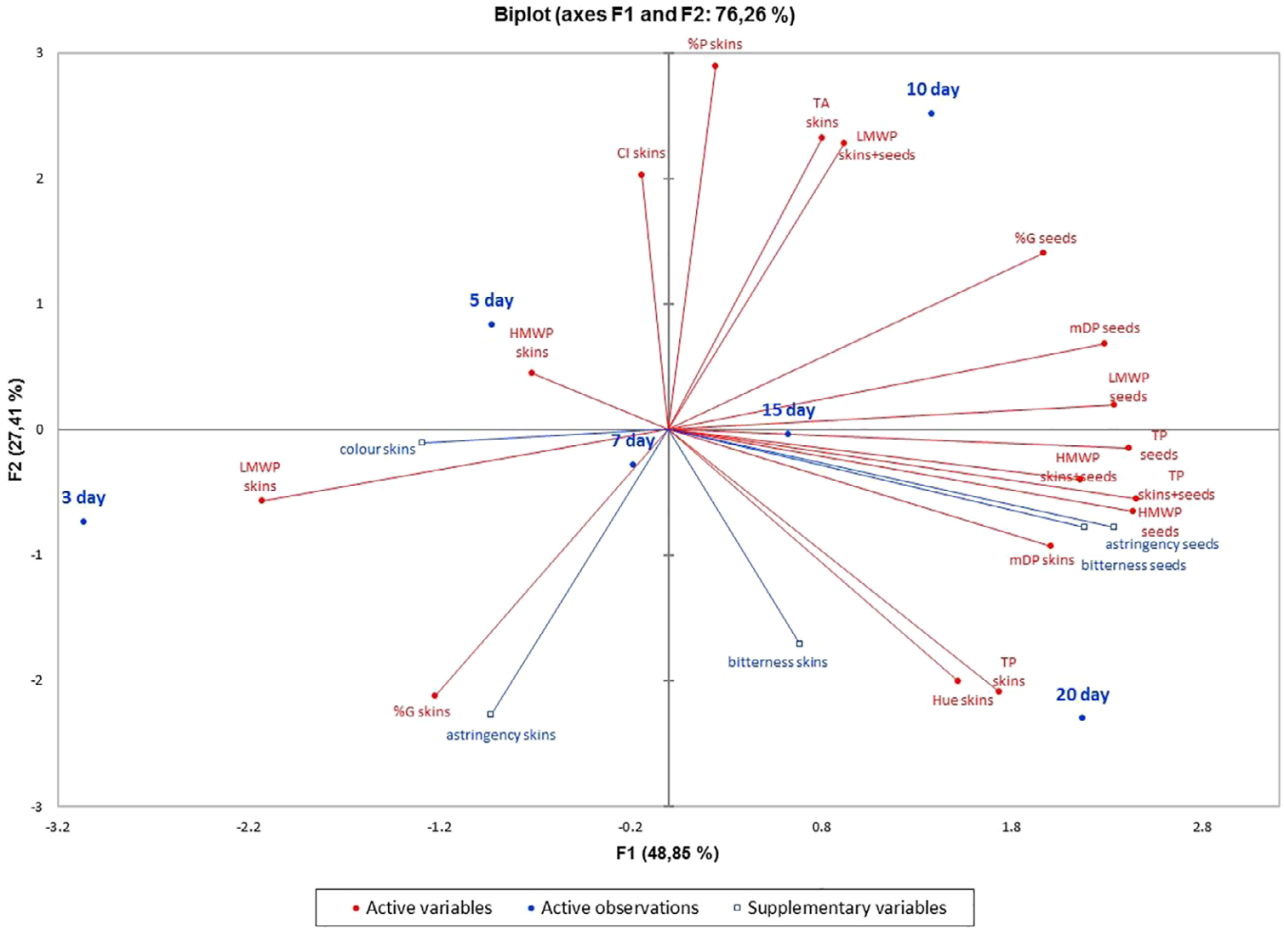
Principal component analysis (PCA) performed on seed and skin wine‐like extracts, time of extraction according to high‐molecular weight proanthocyanidins (HMWPs), low‐molecular weight proanthocyanidins (LMWPs), mean degree of polymerization (mDP), percentage of galloylation (%G), percentage of prodelphinidins (%P), total anthocyanins (TAs), color intensity (CI), total polyphenols (TPs), and sensorial parameters as variables.

During seed tannin extraction there was no significant difference in mDP and %G in extracts from different extraction days (with the exception of the first sampling after 3 days), while the HMWP content increased significantly up to 20 days and the LMWP content reached a plateau after 9 days of extraction. From our observations it could be concluded that higher astringency of seed extracts after longer extraction in wine‐like medium was influenced by the higher content of HMWPs and LMWPs, rather than by structural changes in mDP or %G. Principal component analysis (PCA) showed a positive correlation of HMWP and LMWP content in seed extracts with the astringency and bitterness of seed extracts (Fig. 3). This is in accordance with Landon *et al*.,^15^ who showed that higher astringency has been correlated with higher tannin concentration. However, even if phloroglucynolysis is an appropriate method to assess tannin structure, it does not provide information about the tannin 3D structure that might also influence the perception of astringency.^36^ A sub‐class of the oligomeric cyclic condensed tannins named ‘crown procyanidins’ identified recently in wines^37^ might also have a sensorial impact. Some authors observed that astringency was more affected by the subunit composition of PAs than by the total concentration or the mDP.^38^

Comparing astringency and bitterness in seed and skin extracts, skin PAs were considered on average to be less bitter and astringent than seed PAs. Lower perception of astringency in skin extracts than in seed extracts might be connected with the presence of prodelphinidins in skin PAs and to a lower proportion of galloylation of skin PAs in comparison with seeds. Principal component analysis confirmed a negative correlation between %P and astringency in skin extracts (Fig. 3). This is in accordance with the study by Chira *et al*.^16^

### Phenolic content in fermented wines

The ethanol, total polyphenol, total anthocyanin, color intensity wine hue, LMWP, and HMWP content after different periods of industrial fermentation / maceration is shown in Table 3. Between 3 and 5 days there were non‐significant differences in total phenols, LMWP, and HMWP content. Low alcohol levels in the first 5 days of maceration and cold prefermentation (temperature below 15 °C in the first 2 days) might have affected the initial rate of PAs extraction. High molecular‐weight PAs, LMWPs, and total polyphenol content increased significantly up to 9 days, and thereafter changes in the content were not significant. Fermented wines are a complex medium due to simultaneous extraction of tannins from skins and seeds. High molecular‐weight PAs from skins might therefore affect the significance of ongoing HMWP extraction from seeds. An increase in high polymerized tannin content during extended maceration has also been reported by Vrhovsek *et al*.^22^

**Table 3:** Alcohol content, color attributes, phenolic compounds, and structural composition of proanthocyanidins in fermented wines after different lengths of large‐scale maceration (each day represents the average content in three fermenters)

Day	Ethanol	TP	TA	CI	Hue	LMWP	HMWP	mDP	%P	%G
3	0.6 a	269 a	563 a	2,93 a	0,53 a	387 a	416 a	3.5 a	20.2 a	1.9 a
5	5.7 b	302 a	631 a,b,c	3,07 a	0,55 a	541 a	560 a	3.8 a	28.5 b	5.3 a
7	11.4 c	844 b	1133 b,c,d	4,76 b,c	0,51 a	1321 b	1775 b	5.3 b	18.8 a	23.1 b
9	12.7 c,d	1119 c	1167 d	5,31 b,c	0,51 a	1743 c	2284 b,c	5.9 b	18.1 a	24.9 b
11	13.4 d	1188 c	1113 d	4,87 b	0,54 a	1803 c	2412 c	6.0 b	16,2 a	27.2 b
13	13.8 d	1171 c	994 b	4,20 b,c	0,56 a	1866 c	2673 c	5.8 b	15.6 a	26.9 b
15	14.2 d	1222 c	944 c	4,10 c	0,59 a	1855 c	2789 c	5.5 b	14.7 a	27.3 b

Ethanol in vol.%; TP, total polyphenols as mg L^−1^ of (+)‐catechin; TA, total anthocyanins as mg L^−1^; CI, color intensity; Hue, color hue; LMWPs, low‐molecular weight proanthocyanidins as mg L^−1^ of (+)‐catechin; HMWPs, high‐molecular weight proanthocyanidins as mg L^−1^of cyanidin; mDP, mean degree of polymerization; %P, percentage of prodelphinidins; %G, percentage of galloylation.

Different letters indicate statistically significant differences between extraction days (Tukey's HSD. *P* < 0.05).

The total anthocyanin content was maximal between 7 and 11 days of maceration and decreased after that. Color intensity followed a similar trend, whereas color hue did not change statistically during maceration. The results are consistent with those obtained by others, who have reported that anthocyanin extraction from Cabernet Sauvignon grapes began early in the fermentation process and diminished as fermentation progressed.

### Structural characteristics of proanthocyanidins in fermented wines

Structural analysis of PAs showed that mDP and %G were significant lower and %P significantly higher after 7 days of maceration than after 3 and 5 days. However, there were no significant differences in mDP, %G, and %P after 7, 9, 11, 13, and 15 days of maceration. The mDP of fermented wines was 5.3 after 7 days, 6.0 after 11 days, and 5.5 after 15 days of maceration. A decrease in mDP and increase in %G in the case of longer maceration has been reported by others.^23^, ^39^ The mDPs were 7.0, 8.0 and 5.7 and %Gs were 1.6, 1.8 and 2.5, respectively, after 5, 10, and 20 days of maceration of Cabernet Sauvignon wines.^23^ The significant increase in total polyphenols, LMWPs, HMWPs, and %G, together with the decrease in both mDP and %P subsequently, showed the seed extraction trends after 7 days of maceration. At this time the alcohol level was 11.4%, i.e. 80% of final alcohol.

### Sensory characterization of fermented wines

**Figure 4:**
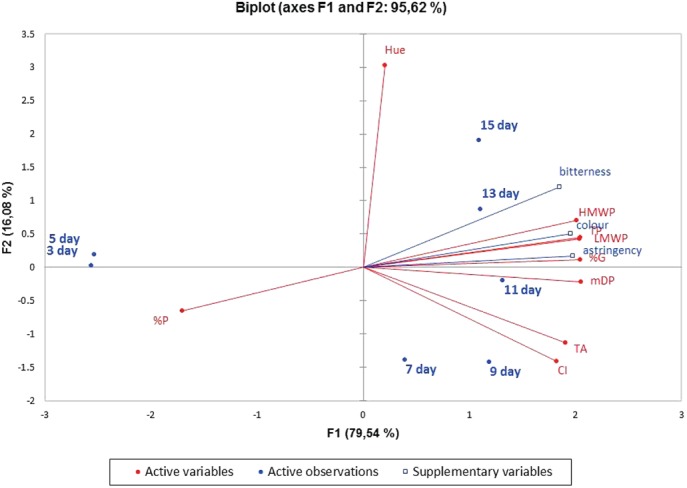
Principal Component Analysis (PCA) performed following large‐scale maceration and time of maceration according to high‐molecular weight proanthocyanidins (HMWPs), low‐molecular weight proanthocyanidins (LMWPs), mean degree of polymerisation (mDP), percentage of galloylation (%G), percentage of prodelphinidins (%P), total anthocyanins (TAs), colour intensity (CI), total polyphenols (TPs) and sensorial parameters as variables.

The sensory characteristics of fermented wines evaluated with rank sums are presented in Table 4. As regards color intensity, the highest ranks for color intensity was after 11 days of maceration; however, there were no significant differences between 9, 11, 13, and 15 days. After 3 and 5 days of maceration, color intensity was ranked lower than in later days. Astringency and bitterness were significantly lower after 3 and 5 days' maceration than after 11, 13, and 15 days. The differences in astringency and bitterness for other days were not significant. It has to be considered that sensory characterization of astringency and bitterness in fermented wines might be influenced by changes in alcohol and reducing sugar content during fermentation. The sensory characterization of skin and seed wine‐like extracts was therefore performed under uniform conditions. The PCA of fermented wines showed a positive correlation between astringency and %G, mDP, HMWP, LMWP, and total polyphenol content in fermented wines and a negative correlation between astringency and %P (Fig. 4).

**Table 4:** Ranking totals (n = 7 tasters, intensity scale 0–7 points) for sensory attributes regarding color, astringency and bitterness of fermented wines on different maceration days

Days of maceration	Color intensity	Astringency	Bitterness
3	8 a^a^	10 a	7 a
5	9 a	14 a,b	10 a,b
7	18 a,b	23 a,b,c	17 a,b
9	25 b,c	24 b,c	21 b,c
11	33 c	24 c,d	30 c
13	26 b,c	28 d	32 c
15	28 b,c	24 c,d	30 c

Different letters indicate statistically significant differences between extraction days (LSD 5%).

### Relationship between extraction time, phenolic content analytical data, and the structural characteristics of PAs

Principal component analysis was carried out to represent graphically the relationship between extraction time, analytical data on phenolic content, and the structural characteristics of PAs in wine‐like extracts (Fig. 3) and during industrial maceration (Fig. 4).

In the case of skin and seed wine‐like extracts, the first two principal components accounted for 76.26% of total variation (F1 + F2) (Fig. 3). The HMWP seeds (mg kg^−1^), TP seeds (mg kg^−1^), and LMWP seeds (mg kg^−1^) were positively correlated with the astringency and bitterness of seed extracts. High Pearson's correlation coefficients confirmed these positive linear relationships (*P* < 0.05). The astringency of seed extracts was correlated with HMWP seeds (R = 0.982), TP seeds (R = 0.942), and LMWP seeds (R = 0.937) but the astringency of skin extracts was negatively correlated with %P (R = −0.813) (Table S2).

In the case of industrial maceration, the first two principal components accounted for 95.62% of total variation (Fig. 4). The HMWP, TPs, LMWP, %G, mDP, TAs and color intensity were highly correlated with the first principal component, which explained 79.54% of total variation. Pearson's correlation coefficients confirmed a high positive linear relationship (*P* < 0.05) of fermented wines' astringency and %G (R = 0.972), LMWP (mg L^−1^) (R = 0.962), mDP (R = 0.958), HMWP (mg L^−1^) (R = 0.952), and TP (mg L^−1^) (R = 0.945), and a negative relationship with %P (R = −0,713) (Table S3). The data obtained are in agreement with Chira *et al*.,^32^ who reported that an increase in mDP and %G increased the perceived astringency; Landon *et al*.,^15^ who showed a positive correlation between astringency and tannin concentration, and Fernández *et al*.^40^ and Vidal *et al*.,^14^ who reported that the presence of prodelphinidins decreased the perception of astringency.

## CONCLUSIONS

During the red wine maceration / fermentation process it is difficult to establish whether tannins in wines come from the grape skins or seeds. The skins and seeds were therefore extracted separately in a wine‐like medium. The low and high molecular‐weight fraction of skin tannins was extracted very rapidly. On the other hand, the high molecular‐weight fraction of seed tannins increased continuously. Monomers and low molecular‐weight seed tannins reached a plateau earlier. The most significant finding in this study was that, during longer seed maceration, an increase in high molecular weight tannin content was more evident than changes in tannin structural characteristics such as the mean degree of polymerization (mDP). The mDP of tannins from skins and seeds was unaffected by the extension of maceration. During industrial maceration, HMWPs and LMWPs increased until 12.7% alcohol was reached; thereafter the increase was not significant, whereas mDP, %G and %P did not change significantly after 11.4% alcohol was reached. It was concluded that, with a longer maceration time, the increase in high molecular weight tannin content was more evident than changes in tannin structural characteristics evaluated by phloroglucynolysis. The high molecular weight tannin fraction from seeds extracted after lengthy (post alcoholic) maceration is important in determining sensorial properties (astringency and bitterness) of red wines. The modification in the composition of seed proanthocyanidins by viticultural practices could account for a change in both their extractability and sensorial properties. Additional research on manipulating the highly polymerized seed tannin fraction (during grape ripening and maceration) is therefore needed.

## Supporting information


**Appendix S1**: Supporting Information
