# Supplementary material for: Effect of extraction time on content, composition and sensory perception of proanthocyanidins in wine‐like medium and during industrial fermentation of Cabernet Sauvignon
Source: J Sci Food Agric. 2020 Feb 4;100(5):1887–96. doi: 10.1002/jsfa.10189 (PMC7079243; doi:10.1002/jsfa.10189)
Supplement: Supplementary file 1 — Appendix S1: Supporting Information [file JSFA-100-1887-s001.docx]

**Supplementary material**

| **Table S1**. Standard deviations of repeatability and reproducibility of the UHPLC-DAD-MS/MS analysis of flavan 3-ols in Merlot wine. | | | |
| --- | --- | --- | --- |
| Matrix | mDP | % G | % P |
| Means of the levels | 4.64 | 15.93 | 17.89 |
| Standard deviation of repeatability of the level (*s_r_*) | 0.05 | 0.35 | 0.22 |
| Standard deviation of reproducibility of the level (*s_R_*) | 0.16 | 0.84 | 0.63 |
| Repeatability limit (*r*) | 0.16 | 1.13 | 0.71 |
| Reproducibility limit (*R*) | 0.51 | 2.68 | 2.03 |
| Uncertainty of repeatability (*U_r_*) | 0.11 | 0.80 | 0.50 |
| Uncertainty of reproducibility (*U_R_*) | 0.36 | 1.90 | 1.44 |
| mDP, The mean degree of polymerisation; %G, the percentage of galloylation; %P, the percentage of prodelphinidins. | | | |

**UHPLC-DAD-MS/MS chromatograms**
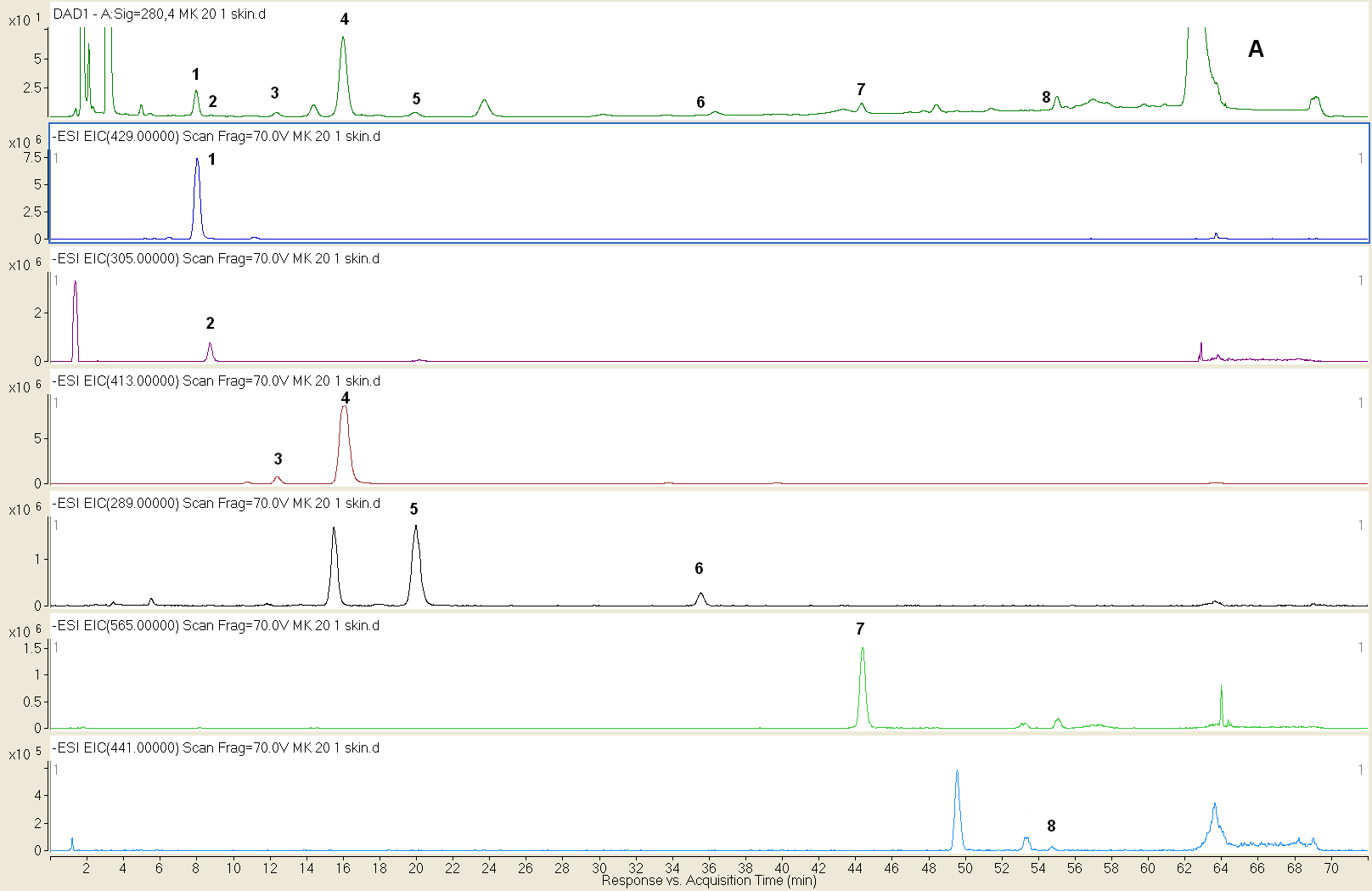


**

**

**Figure S1:** UHPLC-DAD-MS/MS chromatograms of skin wine-like extract after phloroglucinolysis (**A**) and of seed wine-like extract after phloroglucinolysis (**B)**. Peak numbering: **1**, epigallocatechin-phloroglucinol; **2**, gallocatechin; **3**, catechin-phloroglucinol; **4,** epicatechin-phloroglucinol**; 5,** catechin**; 6,** epicatechin**; 7,** epicatechin gallate-phloroglucinol**; 8,** epicatechin gallate**.**

**Total polyphenols in skin and seed wine-like extracts**

The total extractable polyphenol content from grape skins in wine-like solution reached a maximum after 20 days of extraction, however the total polyphenol content in skin extracts did not significantly change from the first sampling after 3 days up to sampling at 15 days. The total extractable polyphenol content in seed extracts increased significantly until the 7^th^ day and there were no significant differences thereafter, meaning a plateau was reached. Initially, i.e. after 3 and 5 days of extraction, the total polyphenol content in skin extracts was higher than in seeds. However, from there on it changed and total polyphenol content was higher in seed extracts. From the results obtained it can be concluded that after longer maceration the proportion of seed polyphenols in wines will be higher than that in skins, even if the skins also contain anthocyanins.





**Figure S2:** Average total extractable polyphenol content (in mg/kg of grape fresh weight) after different extraction times for skins (solid symbols ●) and seeds (hollow symbols ○) in wine-like solution.

The error bars represent the standard deviation of three replicates within each observation (n=3). Different letters indicate statistically significant differences between days of extraction (Tukey's HSD, P<0.05).

**Distribution of extractable low molecular and high molecular weight proanthocyanidins in seeds and skins after different periods of extraction in wine-like solution.**

**Figure S3:** Average distribution (n=3 for each day of extraction) of extractable low molecular weight proanthocyanidins (LMWP) in seeds and skins after different periods of separate extraction in wine-like solution.

**Figure S4:** Average distribution (n=3 for each day of extraction) of extractable high molecular weight proanthocyanidins (HMWP) in seeds and skins after different periods of separate extraction in wine-like solution.

**Table S2**

Pearson correlation matrix for seed and skin wine-like extracts.

| **Variables** | **TA skins** | **TP skins** | **TP seeds** | **TP skins+seeds** | **LMWP skins** | **LMWP seeds** | **LMWP skins+**  **seeds** | **HMWP skins** | **HMWP seeds** | **HMWP skins+**  **seeds** | **CI skins** | **Hue skins** | **mDP skins** | **%G skins** | **%P skins** | **mDP seeds** | **%G seeds** | **colour skins** | **astri-ngency skins** | **bitte-rness skins** | **astri-ngency seeds** | **bitte-rness seeds** |
| --- | --- | --- | --- | --- | --- | --- | --- | --- | --- | --- | --- | --- | --- | --- | --- | --- | --- | --- | --- | --- | --- | --- |
| TA skins | **1** | -0,244 | 0,219 | 0,142 | -0,424 | 0,221 | **0,999** | 0,400 | 0,098 | 0,395 | 0,356 | -0,287 | 0,166 | -0,598 | **0,816** | 0,276 | 0,443 | -0,461 | **-0,994** | -0,321 | -0,043 | 0,113 |
| TP skins | -0,244 | **1** | 0,463 | 0,621 | -0,459 | 0,374 | -0,211 | 0,067 | 0,664 | 0,778 | -0,321 | **0,964** | 0,744 | 0,335 | -0,653 | 0,353 | 0,151 | -0,108 | 0,239 | 0,279 | 0,636 | 0,641 |
| TP seeds | 0,219 | 0,463 | **1** | **0,982** | -0,641 | **0,968** | 0,266 | -0,525 | **0,965** | 0,681 | -0,276 | 0,626 | 0,669 | -0,550 | 0,094 | 0,805 | 0,636 | -0,653 | -0,291 | 0,519 | **0,942** | **0,910** |
| TP skins+seeds | 0,142 | 0,621 | **0,982** | **1** | -0,664 | **0,935** | 0,191 | -0,450 | **0,994** | 0,767 | -0,312 | 0,758 | 0,749 | -0,415 | -0,055 | 0,787 | 0,594 | -0,600 | -0,207 | 0,518 | **0,968** | **0,941** |
| LMWP skins | -0,424 | -0,459 | -0,641 | -0,664 | **1** | -0,597 | -0,454 | -0,113 | -0,640 | -0,784 | -0,339 | -0,429 | -0,802 | 0,293 | -0,185 | **-0,853** | **-0,864** | 0,232 | 0,455 | 0,143 | -0,594 | -0,433 |
| LMWP seeds | 0,221 | 0,374 | **0,968** | **0,935** | -0,597 | **1** | 0,266 | -0,608 | **0,934** | 0,588 | -0,123 | 0,536 | 0,506 | -0,685 | 0,204 | **0,860** | 0,711 | -0,512 | -0,280 | 0,384 | **0,937** | **0,880** |
| LMWP skins+seeds | **0,999** | -0,211 | 0,266 | 0,191 | -0,454 | 0,266 | **1** | 0,374 | 0,147 | 0,430 | 0,338 | -0,247 | 0,202 | -0,613 | 0,807 | 0,313 | 0,469 | -0,487 | **-0,996** | -0,293 | 0,006 | 0,159 |
| HMWP skins | 0,400 | 0,067 | -0,525 | -0,450 | -0,113 | -0,608 | 0,374 | **1** | -0,458 | 0,217 | 0,314 | -0,171 | 0,127 | 0,449 | 0,030 | -0,357 | -0,183 | 0,171 | -0,338 | -0,554 | -0,578 | -0,452 |
| HMWP seeds | 0,098 | 0,664 | **0,965** | **0,994** | -0,640 | **0,934** | 0,147 | -0,458 | **1** | 0,768 | -0,295 | 0,792 | 0,719 | -0,402 | -0,093 | 0,787 | 0,585 | -0,533 | -0,155 | 0,487 | **0,982** | **0,955** |
| HMWPs skins+seeds | 0,395 | 0,778 | 0,681 | 0,767 | -0,784 | 0,588 | 0,430 | 0,217 | 0,768 | **1** | -0,097 | 0,747 | **0,881** | -0,118 | -0,080 | 0,606 | 0,511 | -0,462 | -0,413 | 0,135 | 0,662 | 0,723 |
| CI skins | 0,356 | -0,321 | -0,276 | -0,312 | -0,339 | -0,123 | 0,338 | 0,314 | -0,295 | -0,097 | **1** | -0,473 | -0,239 | -0,298 | 0,549 | 0,319 | 0,556 | 0,603 | -0,302 | **-0,935** | -0,262 | -0,457 |
| Hue skins | -0,287 | **0,964** | 0,626 | 0,758 | -0,429 | 0,536 | -0,247 | -0,171 | 0,792 | 0,747 | -0,473 | **1** | 0,750 | 0,210 | -0,635 | 0,417 | 0,168 | -0,264 | 0,259 | 0,490 | 0,778 | 0,781 |
| mDP skins | 0,166 | 0,744 | 0,669 | 0,749 | -0,802 | 0,506 | 0,202 | 0,127 | 0,719 | **0,881** | -0,239 | 0,750 | **1** | 0,096 | -0,275 | 0,559 | 0,441 | -0,495 | -0,216 | 0,333 | 0,641 | 0,601 |
| %G skins | -0,598 | 0,335 | -0,550 | -0,415 | 0,293 | -0,685 | -0,613 | 0,449 | -0,402 | -0,118 | -0,298 | 0,210 | 0,096 | **1** | **-0,817** | -0,605 | -0,652 | 0,347 | 0,622 | 0,067 | -0,395 | -0,395 |
| %P skins | **0,816** | -0,653 | 0,094 | -0,055 | -0,185 | 0,204 | 0,807 | 0,030 | -0,093 | -0,080 | 0,549 | -0,635 | -0,275 | **-0,817** | **1** | 0,275 | 0,479 | -0,200 | **-0,813** | -0,419 | -0,142 | -0,105 |
| mDP seeds | 0,276 | 0,353 | 0,805 | 0,787 | **-0,853** | **0,860** | 0,313 | -0,357 | 0,787 | 0,606 | 0,319 | 0,417 | 0,559 | -0,605 | 0,275 | **1** | **0,954** | -0,183 | -0,315 | -0,035 | 0,807 | 0,607 |
| %G seeds | 0,443 | 0,151 | 0,636 | 0,594 | **-0,864** | 0,711 | 0,469 | -0,183 | 0,585 | 0,511 | 0,556 | 0,168 | 0,441 | -0,652 | 0,479 | **0,954** | **1** | -0,081 | -0,467 | -0,283 | 0,594 | 0,383 |
| colour skins | -0,461 | -0,108 | -0,653 | -0,600 | 0,232 | -0,512 | -0,487 | 0,171 | -0,533 | -0,462 | 0,603 | -0,264 | -0,495 | 0,347 | -0,200 | -0,183 | -0,081 | **1** | 0,534 | -0,689 | -0,415 | -0,619 |
| astringency skins | **-0,994** | 0,239 | -0,291 | -0,207 | 0,455 | -0,280 | **-0,996** | -0,338 | -0,155 | -0,413 | -0,302 | 0,259 | -0,216 | 0,622 | **-0,813** | -0,315 | -0,467 | 0,534 | **1** | 0,241 | -0,015 | -0,162 |
| bitterness skins | -0,321 | 0,279 | 0,519 | 0,518 | 0,143 | 0,384 | -0,293 | -0,554 | 0,487 | 0,135 | **-0,935** | 0,490 | 0,333 | 0,067 | -0,419 | -0,035 | -0,283 | -0,689 | 0,241 | **1** | 0,480 | 0,581 |
| astringency seeds | -0,043 | 0,636 | **0,942** | **0,968** | -0,594 | **0,937** | 0,006 | -0,578 | **0,982** | 0,662 | -0,262 | 0,778 | 0,641 | -0,395 | -0,142 | 0,807 | 0,594 | -0,415 | -0,015 | 0,480 | **1** | **0,917** |
| bitterness seeds | 0,113 | 0,641 | **0,910** | **0,941** | -0,433 | **0,880** | 0,159 | -0,452 | **0,955** | 0,723 | -0,457 | 0,781 | 0,601 | -0,395 | -0,105 | 0,607 | 0,383 | -0,619 | -0,162 | 0,581 | **0,917** | **1** |
| *Values in bold are different from 0 with a significance level alpha=0,05; LMWP:low molecular weight proanthocyanidins, HMWP: high molecular weight proanthocyanidins; CI: colour intensity; mDP: mean degree of polymerisation; %G: percent of galloylation; %P: percent of prodelphinidins; TA: total anthocyanins; TP: total polyphenols.* | | | | | | | | | | | | | | | | | | | | | | |

**Table S3**

Pearson correlation matrix for fermented wines.

| Variables | TA | TP | LMWP | HMWP | CI | Hue | mDP | %G | %P | colour | astringency | bitterness |
| --- | --- | --- | --- | --- | --- | --- | --- | --- | --- | --- | --- | --- |
| TA | **1** | **0,852** | **0,856** | **0,810** | **0,983** | -0,253 | **0,938** | **0,915** | -0,637 | **0,800** | **0,875** | 0,679 |
| TP | **0,852** | **1** | **0,998** | **0,993** | **0,809** | 0,240 | **0,972** | **0,977** | **-0,831** | **0,967** | **0,945** | **0,943** |
| LMWP | **0,856** | **0,998** | **1** | **0,993** | **0,810** | 0,238 | **0,976** | **0,980** | **-0,804** | **0,958** | **0,962** | **0,945** |
| HMWP | **0,810** | **0,993** | **0,993** | **1** | 0,752 | 0,322 | **0,947** | **0,971** | **-0,830** | **0,943** | **0,952** | **0,957** |
| CI | **0,983** | **0,809** | **0,810** | 0,752 | **1** | -0,346 | **0,906** | **0,854** | -0,592 | **0,770** | **0,802** | 0,603 |
| Hue | -0,253 | 0,240 | 0,238 | 0,322 | -0,346 | **1** | 0,039 | 0,149 | -0,204 | 0,261 | 0,163 | 0,473 |
| mDP | **0,938** | **0,972** | **0,976** | **0,947** | **0,906** | 0,039 | **1** | **0,979** | -0,753 | **0,941** | **0,958** | **0,882** |
| %G | **0,915** | **0,977** | **0,980** | **0,971** | **0,854** | 0,149 | **0,979** | **1** | **-0,781** | **0,926** | **0,972** | **0,902** |
| %P | -0,637 | **-0,831** | **-0,804** | **-0,830** | -0,592 | -0,204 | -0,753 | **-0,781** | **1** | **-0,794** | -0,713 | **-0,784** |
| colour | **0,800** | **0,967** | **0,958** | **0,943** | **0,770** | 0,261 | **0,941** | **0,926** | **-0,794** | **1** | **0,872** | **0,943** |
| astringency | **0,875** | **0,945** | **0,962** | **0,952** | **0,802** | 0,163 | **0,958** | **0,972** | -0,713 | **0,872** | **1** | **0,902** |
| bitterness | 0,679 | **0,943** | **0,945** | **0,957** | 0,603 | 0,473 | **0,882** | **0,902** | **-0,784** | **0,943** | **0,902** | **1** |
| *Values in bold are different from 0 with a significance level alpha=0,05*  *LMWPs:low molecular weight proanthocyanidins, HMWPs: high molecular weight proanthocyanidins; CI: colour intensity; mDP: mean degree of polymerisation; %G: percent of galloylation; %P: percent of prodelphinidins; TAs: total anthocyanins; TPs: total polyphenols.* | | | | | | | | | | | | |
